# Serum albumin sensitization in children with cow's milk allergy: Clinical relevance to red meat reactions

**DOI:** 10.1111/pai.70157

**Published:** 2025-07-24

**Authors:** Cansu Özdemiral, Ilteber Konuralp, Bulent Enis Sekerel

**Affiliations:** ^1^ Department of Pediatric Allergy, Faculty of Medicine Hacettepe University Ankara Turkey; ^2^ Department of Statistics Middle East Technical University Ankara Turkey

**Keywords:** allergen molecule, Bos d 6, cow's milk, cross‐sensitization, food allergy, red meat, serum albumin

## Abstract

**Introduction:**

Sensitization to serum albumins (SAs) in cow's milk allergy (CMA) may contribute to red meat (RM) allergy. This study evaluated the prevalence of SA sensitization, patterns of cross‐sensitization, and clinical relevance to RM allergy in children with CMA.

**Methods:**

Seventy children with CMA (58 current, 12 resolved) who underwent multiplex microarray testing were assessed for allergen sensitization profiles and reported reactions to RM.

**Results:**

Bos d 6 sensitization was identified in 61.4% of patients, and 18% had been advised to avoid RM. Among Bos d 6–sensitized patients, 39.5% were monosensitized. Co‐sensitization rates were 46.5% for Sus s 1, 25.5% for Equ c 3, 23.2% for Fel d 2, and 16.2% for Can f 3. A strong correlation was observed between Bos d 6 and Sus s 1 (*r* = .79), with moderate correlations to Equ c 3 and Can f 3. Hierarchical clustering grouped Bos d 6 with Sus s 1, Equ c 3, and Can f 3. While milk allergen sensitization decreased in the resolved‐CMA group, Bos d 6 persisted in some patients. Although all children tolerated well‐cooked RM, 10% reported allergic reactions via various exposure routes, including one patient with resolved‐CMA. All RM‐reactive patients were sensitized to Bos d 6.

**Conclusions:**

Bos d 6 sensitization is common in children with CMA, yet clinical reactivity to RM is infrequent. Cultural and culinary practices may shape exposure and symptom expression, though avoidance behaviors remain common. Integrating allergen profiles into clinical care may guide risk stratification/avoidance recommendations.

**Abbreviations**
BSA: bovine serum albumin
CCD: carbohydrate determinant inhibitor
CMA: cow's milk allergy
DBPCFC: double‐blind placebo‐controlled food challenge
HC: hierarchical clustering
IgE: immunoglobulin E
IQR: interquartile range
kU_A_/L: kilounits of allergen‐specific IgE per liter
RM: red meat
SA: serum albumin

**Key message**

Bos d 6 sensitization affects approximately 60% of children with CMA and may persist even after clinical resolution. It can underlie allergic reactions to raw red meat through various exposure routes and may also drive cross‐sensitization to other mammalian serum albumins, including Sus s 1, Equ c 3, and Can f 3.

## 1 INTRODUCTION

Cow's milk allergy (CMA) is one of the most common food allergies in children worldwide, affecting 2%–4.5% of infants.^1^, ^2^, ^3^ It primarily results from sensitization to milk proteins such as casein, β‐lactoglobulin, α‐lactalbumin, and bovine serum albumin (BSA). Bovine serum albumin, a whey protein, plays a central role in CMA and has also been implicated in red meat (RM) allergy in certain patients.^4^ However, reliable data on the prevalence of RM allergy among children with CMA remain limited, likely due to regional differences in dietary habits, exposure patterns, and diagnostic practices.^5^

Beef and cow's milk share several allergenic proteins, most notably BSA (Bos d 6) and immunoglobulins (Bos d 7).^6^ Serum albumins are highly preserved proteins found across mammals and birds, leading to significant cross‐sensitizations. In humans, albumin is the most abundant plasma protein, with a molecular weight of approximately 68 kDa. It is synthesized in the liver and distributed throughout body tissues—including milk, meat, and epithelial secretions—via transcytosis. Structurally, Bos d 6 exhibits a predominantly α‐helical conformation composed of three homologous domains arranged in a heart‐shaped structure, stabilized by 17 disulfide bonds.^7^ Importantly, serum albumins are heat‐labile; the structural integrity of Bos d 6 begins to degrade above 60°C, reducing its allergenic potential after sufficient cooking.^8^, ^9^ Serum albumin allergens from other animal species have also been well‐characterized, including Gal d 5 (chicken), Fel d 2 (cat), Equ c 3 (horse), Can f 3 (dog), Sus s 1 (pig), and Cav p 4 (guinea pig).^10^, ^11^ While gastrointestinal sensitization is the predominant route for CMA and RM allergy, respiratory tract sensitization has been described in syndromes such as cat‐pork and bird‐egg syndrome, highlighting the potential for cross‐reactivity between animal‐derived allergens.^5^ Milk, dairy products, and RM are staple components of children's diets globally, although consumption varies by region and culinary tradition. Despite its clinical importance, the relationship between CMA and allergic reactivity to RM—particularly regarding serum albumin sensitization—remains poorly understood.

Therefore, this study aimed to investigate the prevalence of serum albumin sensitization in children with CMA and to evaluate its clinical relevance, with a specific focus on allergic reactivity to RM.

## 2 METHODS

### 2.1 Study design and population

This retrospective study was conducted at the Division of Pediatric Allergy, Hacettepe University Faculty of Medicine. Children with a history of CMA who underwent the Allergen Explorer version 2 (ALEX^2^; MacroArray Diagnostics, Vienna, Austria) test—primarily due to suspected polysensitization—were included. The study protocol was approved by the local ethical committee of Hacettepe University (2025/08‐19), and the requirement for informed consent was waived due to the retrospective design.

Cow's milk allergy was diagnosed based on a positive skin prick test (SPT) and/or cow's milk–specific IgE (sIgE) in patients who had a clear history of clinical reaction following cow's milk ingestion or a positive oral food challenge (OFC), in accordance with PRACTALL guidelines.^12^ Additional inclusion criteria were the availability of ALEX^2^ test results and an age range of 0.5–18 years at the time of ALEX^2^ testing. Exclusion criteria included patients younger than 6 months and those undergoing oral immunotherapy (to avoid possible interference by allergen‐specific IgG).

Patients were classified into two groups based on CMA status at the time of ALEX^2^ testing as either being intolerant (present CMA) or tolerant (past CMA) to cow's milk. Tolerance was confirmed by negative OFC and regularly consumed milk or yogurt consumption without symptoms.

### 2.2 Data collection

Medical records were retrospectively reviewed for demographic data (age and sex), history of atopic diseases (asthma, allergic rhinitis, and atopic dermatitis), other food allergies, family history of atopy, SPT results, total IgE levels, and cow's milk‐sIgE levels.

Sensitization profiles from ALEX^2^ testing were recorded for cow's milk allergen molecules (Bos d 4, Bos d 5, Bos d 6, and Bos d 8), the complete milk extract (Bos d milk), and serum albumin allergen molecules from various sources (Gal d 5—chicken, Fel d 2—cat, Can f 3—dog, Equ c 3—horse, and Sus s 1—pig).^13^ Although Gal d 5 is not a mammalian serum albumin, it was included in the study for two primary reasons. First, it is the most common food allergen among patients with cow's milk allergy. Second, it shows only a weak association with other serum albumins, making it a useful internal control for comparison in the analysis.

### 2.3 Red meat reactivity data collection

Information regarding beef consumption (grilled, boiled, smoked beef, pastrami [beef bacon], and raw salami), the degree of cooking, and any allergic reactions was obtained through medical record review during routine outpatient visits or via telephone interviews with parents. Cooking levels were categorized “well‐cooked, rare or less well‐cooked”. Well‐cooked RM is characterized by thorough browning throughout its thickness, while rare RM typically displays a central red area, a pink peripheral region, and browned outer edges. “Less‐well‐cooked” RM exhibited a central region of pink meat without discernible “red” meat.^14^

### 2.4 Skin prick testing and specific IgE measurements

In our routine practice, skin prick tests (SPT) were performed on the upper back or volar surface of the forearm (for patients older than 5 years) using cow's milk. The extracts were taken from a well using a SPT device (Oryum, Gaziantep, Türkiye). Wheal diameters were measured after 15 min, calculating the mean of the largest diameter and its perpendicular. The procedure was performed by trained technicians with annual quality assessments (requiring a coefficient of variation of <20%.).^15^, ^16^ Serum specific‐IgE levels were determined utilizing the ImmunoCAP method (Thermo Fisher Scientific, Waltham, MA, USA). The following cutoff values were applied: ≥3 mm for SPT, ≥0.35 kU/L for sIgE, ≥0.30 kU_A_/L for sIgE positivity to serum albumin allergen molecules.

### 2.5 Alex^2^ test

The Alex^2^ multiplex microarray panel consists of 178 molecular allergens and 117 allergen extracts, utilizing nano‐bead‐bound allergens immobilized on a nitrocellulose membrane. Patient serum (0.5 mL), diluted 1:5, was incubated on the chip for 2 h. The assay includes CCD inhibitors with ≥85% inhibition efficiency to minimize cross‐reactive carbohydrate determinant (CCD) interference. Allergen sensitizations were detected using alkaline phosphatase‐labeled anti‐human IgE antibodies. The dynamic measurement range of the Alex^2^ assay is between 0.10 and 50 kU_A_/L.^13^

### 2.6 Statistics

Statistical analyses were performed using IBM SPSS Statistics, Version 25.0 (IBM Inc., Armonk, NY). Data were not normally distributed and, therefore, are presented as medians and interquartile ranges (IQR). Differences in nominal variables were tested using chi‐squared or Fisher's exact tests. Correlations between continuous variables were assessed using Spearman's rank correlation test and visualized as heatmaps. The relationship between IgE recognition profiles was analyzed by using the Python programming language version 3.8. Allergenic molecules with a similar pattern of IgE recognition were grouped as hierarchical clusters and presented as a dendrogram. The *p* value of <.05 was considered statistically significant.

## 3 RESULTS

### 3.1 Patient characteristics

Between January 2020 and January 2024, a total of 603 children underwent the ALEX^2^ testing for various allergen sensitizations. Among them, 70 children (11.6%) met the study criteria for CMA, with a median age of 2 years [IQR 1–3.25], and 68.6% were male. This cohort represents approximately 30% of the total cow's milk–allergic population treated at our center. Age distribution was as follows: 6 months–1 year (*n* = 34, 48.6%), 2–3 years (*n* = 19, 27.1%), and 4–13 years (*n* = 17, 24.3%) (Table 1). In addition to CMA, 53 (75.7%) and 62 patients (88.5%) had coexisting hen's egg allergy and at least one other food allergy, respectively.

**TABLE 1:** Characteristics of the patients and serum albumin allergen molecule sensitizations according to age subgroups.

	Entire group	Age subgroups			
		6 months‐1 age group	2–3 age group	4–13 age group	*p*
	*n* = 70	*n* = 34 (48.6%)	*n* = 19 (27.1%)	*n* = 17 (24.3%)	
Age (year) median (IQR)	2 (1–3.25)	1 (1–1)	2 (2–3)	6 (4.5–8.5)	.00
Sex male, *n* (%)	48 (68.6)	27 (79.4)	12 (63.1)	9 (52.9)	.137
Total IgE (kU/L)(k median (IQR)	510 (174–1370)	195 (95–532)	245 (91–991)	674 (282–1514)	.029
Bos d 6					
*n* (%)	43 (61.4)	22 (64.2)	9 (47.4)	12 (70.6)	.315
Median(IQR)	1.35 (0–6.01)	1.11 (0–6.6)	0.2 (0–7.68)	1.71 (0.23–5.30)	.758
Number of SA AM sensitizations					
Mean ± SD	1.54 ± 1.63	0.85 ± 1.32	0.63 ± 1.01	1.41 ± 1.76	.443
Median (IQR)	1 (0–2)	0 (0–1)	0 (0–1)	1 (0–3)	.443
Sus s 1					
*n* (%)	21 (30)	11 (32.4)	5 (26.3)	5 (29.4)	.899
Median (IQR)	0 (0–0.69)	0 (0–0.96)	0 (0–0.31)	0 (0–2.58)	.899
Fel d 2					
*n* (%)	11 (15.7)	5 (14.7)	2 (10.5)	4 (23.5)	.555
Median (IQR)	0 (0–0)	0 (0–0)	0 (0–0)	0 (0–0.22)	.577
Gal d 5					
*n* (%)	15 (21.4)	8 (23.5)	1 (5.3)	6 (35.3)	.086
Median (IQR)	0 (0–0.12)	0 (0–0.25)	0 (0–0)	0 (0–0.81)	.302
Can f 3					
*n* (%)	7 (10)	2 (5.9)	1 (5.3)	4 (23.5)	.105
Median (IQR)	0 (0–0)	0 (0–0)	0 (0–0)	0 (0–0.33)	.187
Equ c 3					
*n* (%)	12 (17.1)	4 (11.8)	3 (15.8)	5 (29.4)	.341
Median (IQR)	0 (0–0)	0 (0–0)	0 (0–0)	0 (0–0.49)	.430

Abbreviations: AM, allergen molecule; CM, cow's milk; IQR, interquartile range; SA, serum albumin; SD, standard deviation.

Atopic dermatitis was present in 80.0% of patients, while asthma and allergic rhinitis were each reported in 31.4%. A positive family history of allergic diseases was noted in 72.4% of cases. Based on Alex^2^ test, the most common additional sensitizations included grass pollen (23 patients, 32.8%), cypress pollen (20 patients, 28.5%), Artemisia pollen (13 patients, 18.5%), Alternaria mold (6 patients, 8.5%), mites (10 patients, 14.2%), furry animals (25 patients, 35.7%), and other foods (68 patients, 97.1%).

Cow's milk allergy was diagnosed at a median age of 6 months (IQR: 2–4 months). All patients had positive SPT to cow's milk, with a median wheal diameter of 10 mm (range: 5–35 mm; IQR: 8–13 mm). The maximum interval between SPT and ALEX^2^ testing was 6 months.

### 3.2 Sensitization to cow's milk allergen molecules

The sensitization rates for cow's milk allergen molecules were as follows: Bos d 4 (82.8%, *n* = 58), Bos d 8 (81.4%, *n* = 57), Bos d 5 (70.0%, *n* = 49), and Bos d 6 (61.4%, *n* = 43). Interestingly, sensitization to cow's milk extract (Bos d milk) was only observed in 57 of these 70 patients (81.4%) with a median level of 4.68 kU_A_/L (IQR: 0.73–16.9). Among these sensitizations, only Bos d 4 sensitization was significantly associated with the presence of Bos d 6 sensitization (Odds Ratio [OR]: 6.67; 95% Confidence Interval [CI]: 1.61–27.57; *p* = .009). No significant associations were found between Bos d 6 sensitization and concurrent allergic diseases (atopic dermatitis: *p* = .30; asthma: *p* = .49; allergic rhinitis: *p* = .56) via point‐biserial correlation analysis.

### 3.3 Sensitization to serum albumins and their relationships

Sensitization to serum albumins from pig (Sus s 1), horse (Equ c 3), cat (Fel d 2), dog (Can f 3), and egg (Gal d 5) was identified in 21 (30%), 12 (17.1%), 11 (15.7%), 7 (10.0%), and 15 (21.4%) patients, respectively. Among the 53 patients with coexisting hen's egg allergy, sensitization to Gal d 5 was present in 13 of these patients (24.5%).

Twenty‐one patients (30.0%) showed sensitization to a single serum albumin. Among them, only four were negative for Bos d 6, and all four were positive for Gal d 5. No patients were solely sensitized to Fel d 2, Can f 3, Equ c 3, or Sus s 1.

**FIGURE 1:**
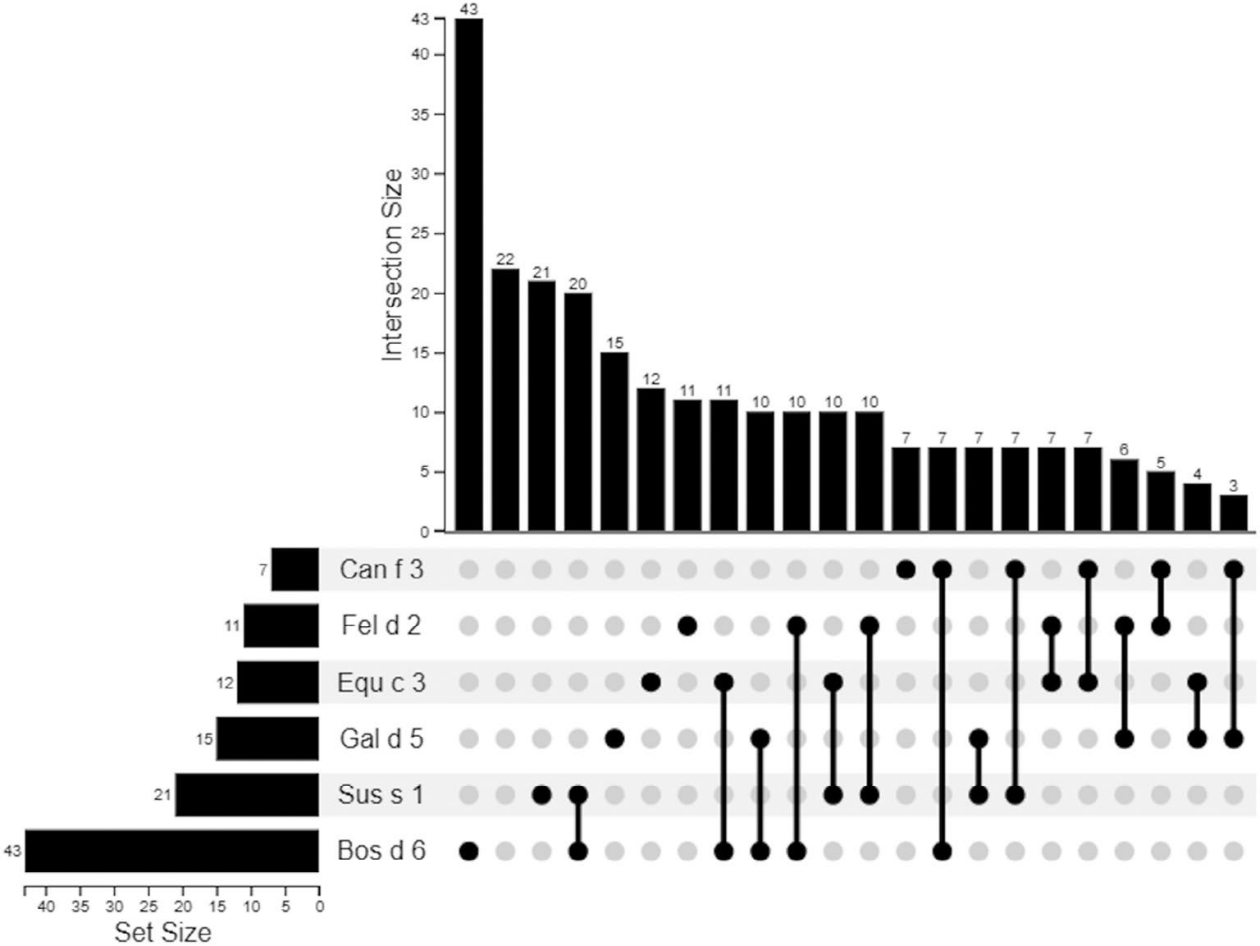
Binary positivity of serum albumins.

Among the 43 Bos d 6–positive patients, 17 (39.5%) exhibited sensitization exclusively to Bos d 6. Sensitization rates to Sus s 1, Equ c 3, Fel d 2, Gal d 5, and Can f 3 were 46.5% (*n* = 20), 25.5% (*n* = 11), 23.2% (*n* = 10), 23.2% (n = 10), and 16.2% (*n* = 7), respectively. Figure 1 displays binary sensitization patterns. Conditional probabilities of serum albumin sensitization given Bos d 6 positivity were as follows: Sus s 1 (47%), Equ c 3 (26%), Fel d 2 (23%), Gal d 5 (23%), and Can f 3 (16%).

**FIGURE 2:**
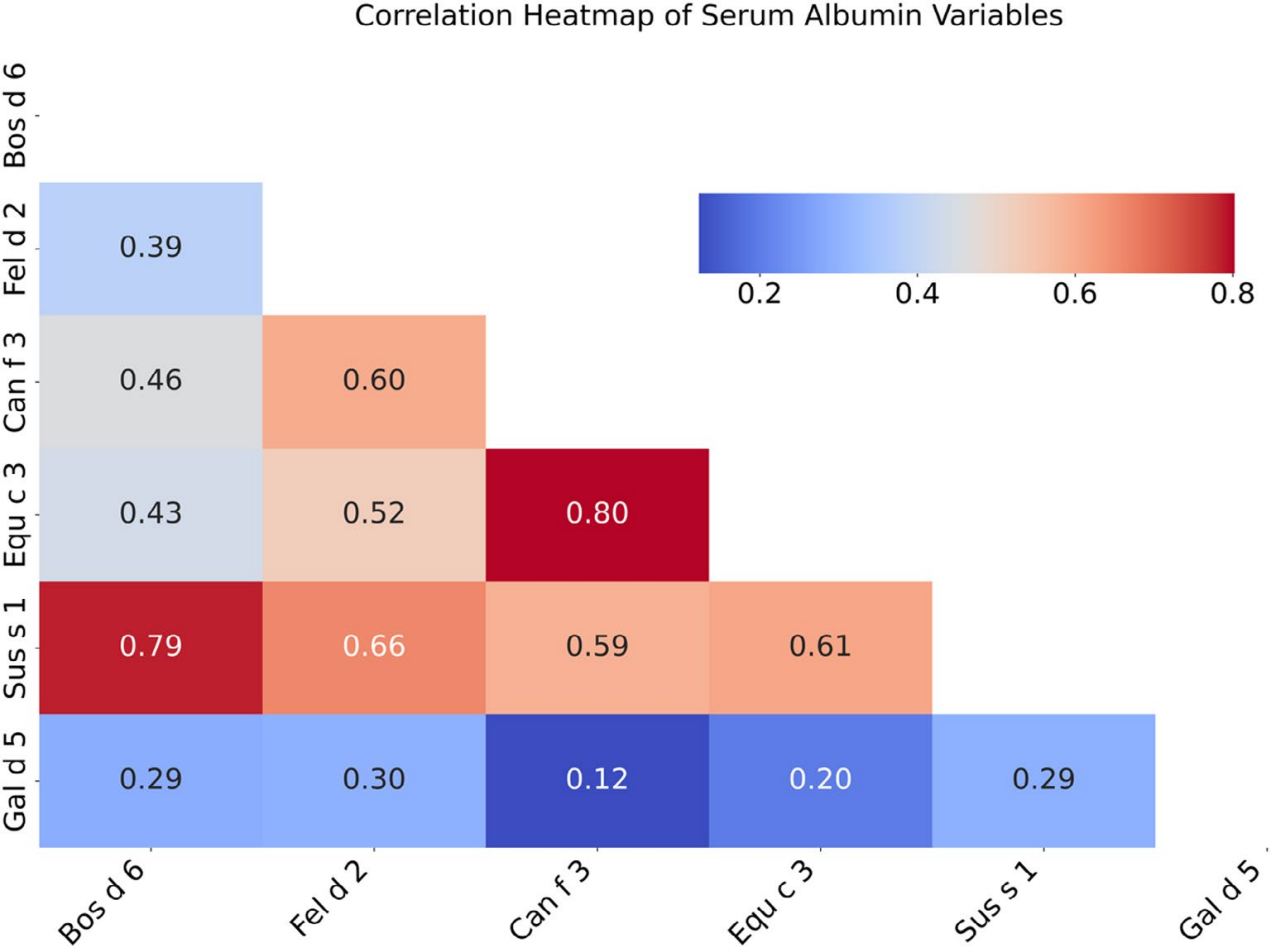
Correlation heatmap of serum albumins.

**FIGURE 3:**
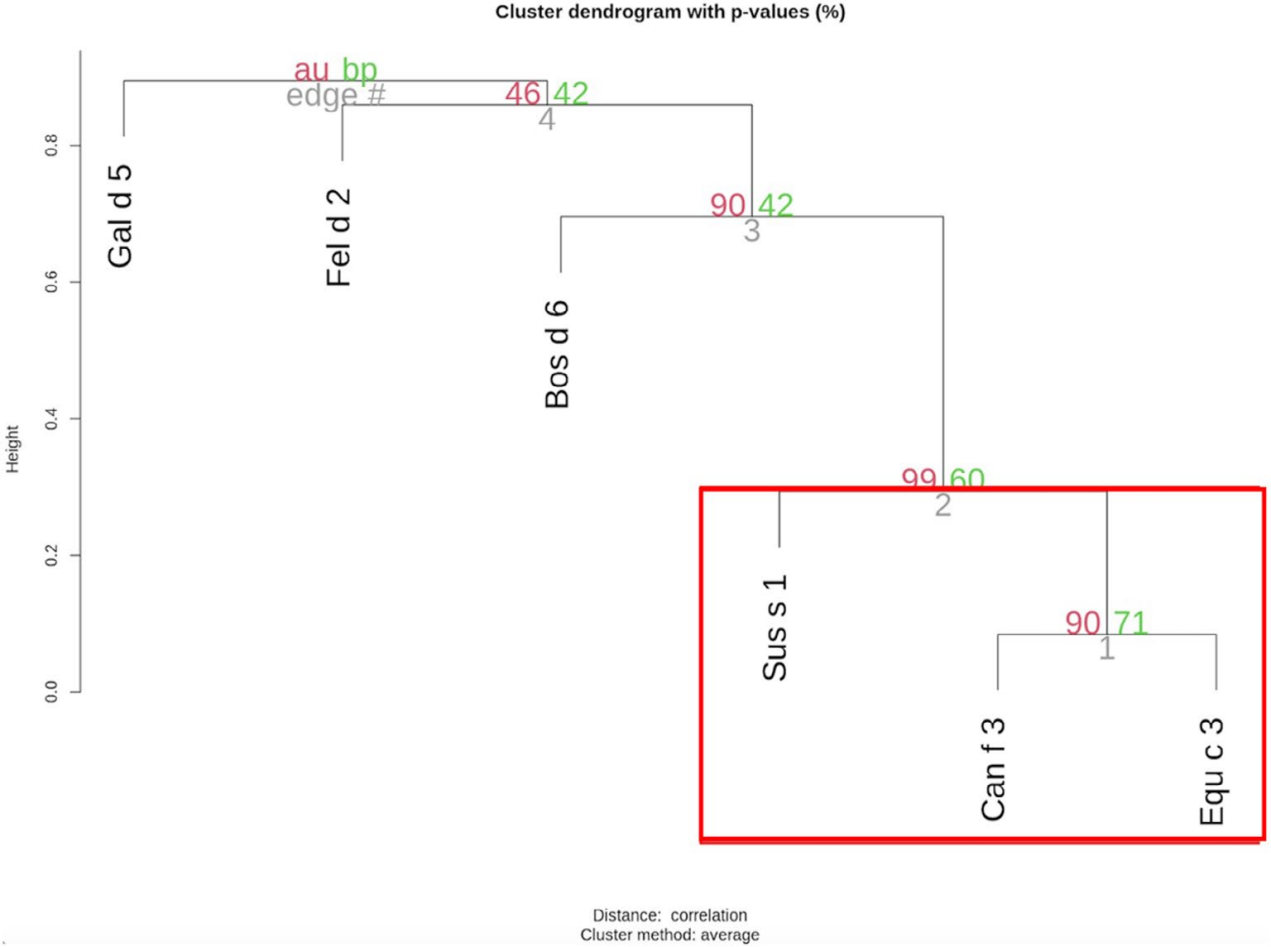
Hierarchical clustering of serum albumins.

Correlation analysis (Figure 2) showed a strong positive correlation between Bos d 6 and Sus s 1 (*r* = .79), and moderate correlations with Can f 3 (*r* = .46) and Equ c 3 (*r* = .43). Hierarchical clustering results, shown in Figure 3, revealed a prominent cluster comprising Equ c 3, Can f 3, and Sus s 1, which was closely associated with Bos d 6. In contrast, Gal d 5 and Fel d 2 exhibited only weak associations with this cluster.

None of the patients sensitized to Fel d 2, Can f 3, or Equ c 3 were co‐sensitized to their corresponding major and genuine animal allergens—Fel d 1, Can f 1/4/5, and Equ c 1/4, respectively.

### 3.4 Subgroup characteristics: Tolerance to cow's milk

Patients were divided based on their CMA status at the time of ALEX^2^ testing: those who had developed tolerance (past CMA, *n* = 12, 17.1%) and those still allergic (present CMA, *n* = 58, 82.8%). Bos d 6 positivity was observed in 35 (60.3%) patients in the present CMA group and in 8 (66.7%) patients in the past CMA group (*p* = .75). In 12 patients with past CMA, co‐sensitization for Bos d 6 and Bos d 8, Bos d 4, and Bos d 5 was observed in 4, 6, and 3 patients, respectively (*p* = .576, .222, and .491). Tolerance to milk occurred at a median age of 35 months (range: 18–87 months; IQR: 21–45 months). Compared to the present CMA group, the past CMA group had significantly fewer patients sensitized to Bos d 4 (*p* = .039), Bos d 5 (*p* = .001), Bos d 8 (p = .001), and Bos d milk (*p* = .007) (Table 2). Although not statistically significant, a reduction in the number of sensitized milk proteins was observed numerically in the past CMA group over time (*p* > .05) (Table S1).

**TABLE 2:** The cow's milk allergen molecule and serum albumin allergen molecule sensitizations according to the status of cow's milk allergy.

	Status of cow's milk allergy		
	Present CMA (*n* = 58)	Past CMA (*n* = 12)	*p*
Age (year); median (IQR)	1 (1–3)	3 (1.25–4.75)	.**049**
Sex male, *n* (%)	42 (72.4)	6 (50)	.174
Total IgE (kU/L) median (IQR)	254 (147–1129)	209 (80–1359)	.542
*n* of CM AM sensitizations			
Mean ± SD	3.13 ± 0.92	1.91 ± 1.24	.**001**
Median (IQR)	3 (3–4)	2 (1–3)	.**001**
Bos d 4			
*n* (%)	50 (86.2)	7 (58.3)	.**039**
Median (IQR)	3.2 (0.86–10.1)	0.42 (0.04–0.79)	**<.001**
Bos d 5			
*n* (%)	45 (77.6)	3 (25.0)	.**001**
Median (IQR)	2.69 (0.36–8.29)	0.05 (0–0.5)	.**001**
Bos d 6			
*n* (%)	35 (60.3)	8 (66.7)	.755
Median (IQR)	1.35 (0–6.94)	1.13 (0.13–3.2)	.818
Bos d 8			
*n* (%)	52 (89.7)	5 (41.7)	.**001**
Median (IQR)	6.77 (1.69–14.75)	0.22 (0–0.85)	**<.001**
Bos d milk			
*n* (%)	51 (87.9)	6 (50)	.**007**
Median (IQR)	6.38 (2.2–20.4)	0.34 (0–0.70)	**<.001**
*n* of SA AM sensitizations			
Mean ± SD	1.05 ± 1.48	0.33 ± 0.65	.108
Median (IQR)	0 (0–1.25)	0 (0–0.75)	.108
Sus s 1			
*n* (%)	20 (34.5)	1 (8.3)	.092
Median (IQR)	0 (0–1.17)	0 (0–0)	.088
Fel d 2			
*n* (%)	10 (17.2)	1 (8.3)	.675
Median (IQR)	0 (0–0)	0 (0–0)	.361
Can f 3			
*n* (%)	7 (12.1)	0	.343
Median (IQR)	0 (0–0)	0 (0–0)	.176
Equ c 3			
*n* (%)	11 (19.6)	1 (8.3)	.673
Median (IQR)	0 (0–0)	0 (0–0)	.327
Gal d 5			
*n* (%)	14 (24.1)	1 (8.3)	.439
Median (IQR)	0 (0–0.24)	0 (0–0.07)	.633

Abbreviations: AM, allergen molecule; CM, cow's milk; IQR: interquartile range; *n*, number; SA, serum albumin; SD, standard deviation.

### 3.5 Characteristics of red meat‐reactive patients

Seven patients (10.0%) reported allergic reactions to RM: ingestion (*n* = 3; beef = 1, raw salami = 2), contact (*n* = 3; raw meat = 2, blood = 1), and inhalation (*n* = 1). All RM‐allergic patients were positive for Bos d 6, accounting for 16.2% of Bos d 6–sensitized individuals. Table 3 presents characteristics and sensitization of red meat‐reactive patients. Thirteen patients (18.5%) were advised by their local physicians to follow a beef elimination diet due to CMA, for a median duration of 1.5 years (range: 0.5–8 years; IQR: 1–3.75 years). These patients tolerated well‐cooked grilled or boiled RM. Eight (11.4%) also tolerated less well‐cooked RM, and none had consumed rare RM.

**TABLE 3:** Characteristics and the sensitization of the patients with red meatallergy.

Pt. No	Incident	CMA status at time of ALEX^2^	Clinical reactivity to red meat	Treatment	Animal/ poultry allergy	Total IgE (ALEX^2^/ImmunoCap)	Bos d milk/sIgE to milk	Bos d meat	Bos d 6	Gal d 5	Fel d 2	Can f 3	Equ c 3	Sus s 1
15	Contact with raw meat	Present CMA	U	–	–	245/−	22.1/−	3.27	**7.74**	0	0.4	0	0	3.12
21	Contact with blood^a^	Present CMA	U, A	Antihistamine	E	186/410	0/9.18	0.38	**10.07**	0	0	0	0	3.08
23	Eating raw salami^b^	Present CMA	U, A	Antihistamine Corticosteroid	E	698/1152	42.7/15.9	0	**8.12**	0	0	0	0	0.31
24	Contact with raw meat	Present CMA	U, A	–	E	491/586	4.1/86.3	2.52	6.70	25.66	1.22	1.20	1.77	6.80
25	Eating raw salami^b^	Present CMA	U, A	Corticosteroid	E	1282/1680	20.0/200	10.30	7.68	0	5.08	**21.15**	4.8**7**	10.22
33	Eating raw beef	Past CMA	U, A, S	Antihistamine^c^	E	77/176	0.52/11.9	0	**2.57**	0	0	0	0	0
66	Inhalation of steam from boiling meat	Present CMA	RD	Short‐acting beta2 agonist	E	1564/2188	30.7/1055	2.44	**29.21**	13.17	0	1.64	0.91	2.49

*Note*: Bold notification represents the highest value in mammalian serum albumins.

Abbreviations: A, angioedema; CMA, cow's milk allergy; E, egg; Pt, patient, RD, respiratory distress; S, syncope; U, urticaria.

^a^ During Eid al‐Adha.

^b^ Not include milk.

^c^ Epinephrine not given; syncope had resolved upon hospital arrival.

Pastrami, raw salami, and smoked meat were consumed by 13 (18.5%), 12 (17.1%), and 8 (11.4%) patients, respectively, with no reported reactions to pastrami or smoked meat. Twenty‐seven patients (38.5%) reported contact with raw meat; two experienced allergic reactions.

The summary characteristics regarding RM‐reactive patients with reference to the exposure histories are outlined below:

One ‐year‐old boy (patient #33) experienced anaphylaxis (syncope, urticaria, and angioedema on hands and face) 15 min after inadvertently consuming raw beef. He was given antihistamine at the hospital and continued to tolerate well‐cooked meat with no RM elimination. Pastrami, smoked beef, and raw salami were subsequently consumed without reaction. A 4‐year‐old patient (#23) had an allergic reaction (urticaria, itching, flushing, and facial angioedema) 5 min after the first consumption of raw salami, resolving with antihistamine and steroid treatment. Another patient (#25) experienced urticaria after the first consumption of raw salami but tolerated well‐cooked RM regularly and subsequently avoided raw salami. A 2‐year‐old boy (#21) who regularly tolerated well‐cooked RM developed urticaria and angioedema on his hands and face after contact with blood during Eid al‐Adha, which improved with antihistamines; he continued to tolerate well‐cooked RM. Two patients (#15 and #24) with no history of reaction to cooked RM experienced reactions (urticaria and angioedema in one, urticaria on hands in the other) after contact with raw RM; both continued to eat cooked RM and avoided raw RM thereafter. A 2‐year‐old boy (Patient #66) experienced bronchospasm after inhaling steam from boiling beef. After a 6‐year beef elimination diet, he could regularly consume boiled or well‐cooked beef. Three patients reported gastrointestinal symptoms (abdominal pain, nausea, or diarrhea) following consumption of excessive amounts of RM.

## 4 DISCUSSION

While serum albumins are not mostly major allergens, they represent a notably intriguing category of allergens. Primarily, they possess sequences analogous (47%–93%) with human serum albumin and, unexpectedly, they can sensitize atopic individuals.^17^ The role of serum albumin in allergic diseases is complex and not fully elucidated. This study presents findings on serum albumin sensitizations, emphasizing Bos d 6 within the context of RM allergy, in a cohort of 70 children with CMA who underwent ALEX^2^ testing.

Bovine serum albumin (Bos d 6) acts as a major allergen in RM and a minor allergen in cow dander and milk.^18^, ^19^, ^20^ In a large cohort of allergic individuals (*n* = 16,408), Bos d 6 sensitization was reported in 3.6% of cases.^21^ Theoretically, Bos d 6 sensitization may develop through two primary pathways: first, as a direct consequence of CMA, and second, through cross‐sensitization to mammalian SAs encountered in the context of animal exposure. While this study primarily focused on the former pathway, we recognize the inherent difficulty in fully distinguishing between these mechanisms—particularly in a setting such as Türkiye, where pet ownership is increasing and the stray animal population is substantial. Nonetheless, our study group showed prominent sensitization (over 80%) to major cow's milk allergens, including Bos d 4, Bos d 5, and Bos d 8, as shown previously in CMA. Furthermore, the absence of concomitant sensitization to the major and genuine allergens of common pets (Fel d 1 and Can f 4/5)^13^ among children with Bos d 6 sensitization supports the effectiveness of our cohort selection in minimizing this potential confounding factor. Restani et al. reported that among 60 children exhibiting immediate reactions to milk, as confirmed by double‐blind placebo‐controlled food challenge (DBPCFC), 61.3% demonstrated the presence of allergen‐specific IgE antibodies to Bos d 6.^22^ However, in another study, Bos d 6 positivity was detected in 3.8% of 78 cow's milk allergic patients, and only one (1.2%) patient was reported to have milk and meat allergy.^23^ This great variance in the prevalences of Bos d 6 sensitization (3.8%–92.9%) across studies likely reflects differences in study populations, diagnostic methods, and regional exposure patterns. While many investigations report rates between 40% and 60%, higher frequencies have been observed in smaller cohorts.^4^, ^24^, ^25^, ^26^, ^27^ In the present study, Bos d 6 sensitization was found in 61.4% of patients, with 39.5% being monosensitized.

The sequence identity among all mammalian serum albumins exceeds 70%, which is the typical threshold for cross‐reactivity.^28^, ^29^ In a study comprising 200 animal hair/dander allergic patients represent 30% of individuals were allergic to serum albumins. Most of the patients allergic to albumins reacted to dog, cat, and horse albumin, which also bound a high percentage of albumin‐specific IgE. Twenty percent of the patients detected bovine and chicken serum albumin.^30^ In a study enrolling cat allergic 85 patients without cow's milk and meat allergy, it was reported that 15 patients had sIgE to Fel d 2, and seven were co‐sensitized to Bos d 6.^31^ In our study, the correlation analysis showed a very strong positive correlation between Can f 3 and Equ c 3. Strong positive correlations were observed between (I) Fel d 2 and Sus s 1, as well as Can f 3, and (II) Sus s 1 and Bos d 6, along with Equ c 3. Hierarchical clustering grouped Bos d 6 with Sus s 1, Equ c 3, and Can f 3.

As egg was the most commonly associated food allergy in our young CMA cohort, consistent with previous findings,^32^ we included Gal d 5 in our analysis, despite its expected limited cross‐reactivity with mammalian serum albumins. Its inclusion also enabled us to assess the internal consistency of our data, highlighting that sensitization to poultry serum albumin differs immunologically from its mammalian counterparts. In line with this, Gal d 5 was clearly separated from other serum albumins in our cluster analysis and exhibited minimal to no cross‐reactivity with the other animal serum albumins evaluated. While prior studies reported a low prevalence of chicken serum albumin‐specific IgE in egg‐allergic patients (2 of 46; 4.3%),^33^ we found a higher sensitization rate, with 24.5% (13 of 53) of egg‐allergic patients in our cohort sensitized to Gal d 5.

Petersen et al. reported significantly difference with using the ImmunoCAP method (Thermo Fisher Scientific, Uppsala, Sweden) between the levels of sIgE to cow's milk, α‐lactalbumin (nBos d 4), β‐lactoglobulin (nBos d 5), and casein (nBos d 8) and the time to remission. This finding leads to a separation of children with persistent CMA from those who outgrow the allergy and the nonallergic group.^34^ We observed that Bos d 4, Bos d 5, and Bos d 8 were significantly decreased in the past CMA group compared to the present CMA group. To our knowledge, this is the first report showing that Bos d 6 sensitization may persist even after clinical tolerance to milk has been achieved (8 out of 12 patients; 66.7%; *p* > .05). However, we also observed that Bos d 6 sensitization tends to gradually resolve over time following the resolution of CMA.

In the literature, 13%–20% of patients with CMA demonstrated RM‐allergy confirmed with oral food challenges.^4^, ^14^, ^35^, ^36^ Also, BSA was suggested to serve as an indicator of CMA in pediatric patients with RM‐allergy.^14^ Twenty‐eight children diagnosed with RM‐allergy performed skin prick tests and a DBPCFC, revealing that 26 (92.9%) were allergic to cow's milk.^4^ In our study involving children with current or past CMA, approximately 60% demonstrated Bos d 6 sensitization, with a small subset reporting a history of allergic reactivity. Notably, all of our RM‐allergic patients exhibited sensitization to Bos d 6.

The epitopic domains responsible for IgE binding can be modified or inactivated by heating or other treatments (homogenization and freeze‐drying) that alter the conformation of the epitopes.^37^ Heat treatment has been demonstrated to alter the allergenicity of beef and BSA, reducing but not completely abolishing their ability to bind IgE from patients.^9^, ^18^, ^38^ On the clinical level, individuals exhibit allergic symptoms to raw meat or demonstrate positive SPT findings for raw meat and BSA; nevertheless, they tolerate cooked meat without responses during oral food challenges and show negative skin prick test results for BSA.^14^, ^39^ In our study, although local physicians recommended beef elimination for 18% of the patients, we observed that all participants tolerated well‐cooked beef—a common preparation in Turkish cuisine. Moreover, patients with prior exposure also tolerated pastrami, a spiced and air‐dried processed meat. This finding may illustrate how culinary and cultural differences in RM consumption can influence the manifestation of allergic reactions.

Tonnel et al. reported two adult patients who have severe acute asthma while cutting raw meat (beef and lamb) but no symptoms after ingestion of cooked meat. One of these patients had similar symptoms after unusual raw meat ingestion and had positive SPT results for serum albumins of beef, pork, horse, dog, and cat.^40^ The sensitization to BSA causes not only symptoms after ingestion but could emerge symptoms with inhalation. Bos d 6 inhalation was reported to have caused two cases of occupational asthma in laboratory workers.^41^, ^42^

Our study also indirectly underscores the value of documenting the full spectrum of IgE‐mediated sensitization using multiplex microarray analysis. Specifically, serum albumin sensitization may result from either cow's milk allergy or animal allergy, and a comprehensive sensitization profile assists in identifying the underlying cause. Moreover, in the context of cow's milk allergy, the presence of Bos d 6 sensitization may indicate an increased risk of allergic reactions to raw red meat. Documenting the complete sensitization profile facilitates the identification of cross‐reactivities, enables risk stratification, and supports personalized management strategies—helping to prevent unnecessary restrictions in polysensitized individuals.

Among those reporting red meat allergy, reactions were limited to specific forms of exposure, that is, contact: two to raw meat, and one to contact with bovine blood; ingestion: two to raw salami and one to raw meat; and one instance via inhalation. The most severe cases involved syncope and bronchospasm following raw meat consumption and inhalation of steam from cooked meat, respectively. Overall, our findings suggest that the risk of clinically significant reactions was low in the majority of Bos d 6–sensitized patients, and most reported reactions were mild and self‐limiting. However, the observation that 18% of patients had adhered to long‐term beef elimination underscores the potential impact of increased awareness and access to information—possibly contributing to heightened anxiety and unnecessary avoidance behaviors.

## 5 CONCLUSION

Bos d 6 sensitization is found in over half of children with CMA and may contribute to RM allergy in some cases. Although this sensitization often declines after CMA resolves, it can persist and cause reactions in a subset of individuals. Bos d 6 sensitization further drives sensitization to other animal serum albumins, particularly to Sus s 1, Equ c 3, and Can f 3. Culinary and cultural practices influence exposure to intact Bos d 6, affecting both the frequency and nature of RM‐related reactions. However, increased awareness of the CMA–RM allergy link may drive avoidance behaviors more than actual reaction risk. Our findings highlight the importance of providing accurate, context‐specific guidance on the risks of different RM preparations rather than focusing solely on Bos d 6 sensitization status. Integrating allergen molecule data, clinical relevance, and allergen stability into routine assessment may improve risk evaluation and support more tailored prevention strategies for children with CMA.

## AUTHOR CONTRIBUTIONS

**Cansu Özdemiral:** Data curation; visualization; writing – original draft; methodology; investigation; formal analysis. **Ilteber Konuralp:** Formal analysis; visualization; writing – review and editing; methodology. **Bulent Enis Sekerel:** Conceptualization; writing – review and editing; project administration; supervision; methodology; investigation.

## FUNDING INFORMATION

This study was not supported by any sponsor or funder.

## CONFLICT OF INTEREST STATEMENT

The authors declare no conflict of interest in relation to this study.

## PEER REVIEW

The peer review history for this article is available at https://www.webofscience.com/api/gateway/wos/peer‐review/10.1111/pai.70157.

## Supporting information


Table S1.
