# Supplementary material for: Serum albumin sensitization in children with cow's milk allergy: Clinical relevance to red meat reactions
Source: Pediatr Allergy Immunol. 2025 Jul 24;36(7):e70157. doi: 10.1111/pai.70157 (PMC12290251; doi:10.1111/pai.70157)
Supplement: Supplementary file 1 — Table S1. [file PAI-36-e70157-s001.docx]

**Supplementary Table.** The cow’s milk allergen molecule sensitizations and serum albumin allergen molecule sensitizations according to duration after diagnosis or resolution

|  | **Present CMA (n=58)** | | | **Past CMA (n=12)** | |
| --- | --- | --- | --- | --- | --- |
| **Duration after diagnosis/resolution** | **<1 year after diagnosis** | **1-2 year after diagnosis** | **>3 year after diagnosis** | **<1 year after resolution** | **>1 year after resolution** |
| **n** | 26 | 16 | 16 | 8 | 4 |
| **Age** (year)  median (IQR)* | 1  (0-1) | 2  (1-2) | 5  (3.2-6.75) | 2  (1-3.75) | 4.5  (3-7.5) |
| **n of CM AM Sensitizations** mean±SD**  median (IQR) | 3.11±1.44  3(2.75-4.0) | 2.87±1.07  3(2-4) | 3.43±0.72  4(3-4) | 2.37±0.91  2(2-3) | 1±1.41  0.5(0-2.5) |
| **Bos d 4**  n (%),  median (IQR) | 21(80.8)  2.54(0.83-5.76) | 13(81.3)  3.83(0.44-10.2) | 16(100)  7.78(1.79-26.6) | 6(75)  0.56(0.2-1.1) | 1(25)  0.11(0.11-0.60) |
| **Bos d 5**  n (%),  median (IQR) | 20(76.9)  2.69(0.3-6.04) | 10(62.5)  1.18(0.04-8.7) | 15(93.8)  6.32(0.91-28.4) | 3(37.5)  0.18(0-0.89) | 0  0(0-0.18) |
| **Bos d 6**  n (%),  median (IQR) | 17(65.4)  1.86(0-7.9) | 8(50)  0.33(0-7.3) | 10(62.5)  1.42(0.05-6.57) | 7(87.5)  1.84(0.52-3.2) | 1(25)  0.15(0.02-3.37) |
| **Bos d 8**  n (%),  median (IQR) | 23(88.5)  3.67(1.51-7.83) | 15(93.8)  8.39(2.59-16.7) | 14(87.5)  9.84(3.94-28.9) | 3(37.5)  0(0-0.94) | 2(50)  0.32(0.18-0.84) |
| **n of SA AM Sensitizations** mean±SD**  median (IQR) | 1.07±1.44  0.5(0-2) | 0.62±1.08  0(0-1) | 1.43±1.78  1(0-3.5) | 0.25±0.46  0(0-0.75) | 0.5±1.0  0(0-1.5) |
| **Sus s 1**  n (%),  median (IQR) | 10(38.5)  0(0-1.8) | 4(25)  0(0.3) | 6(37.5)  0(0-2.62) | 1(12.5)  0(0) | 0  0(0-0.17) |
| **Fel d 2**  n (%),  median (IQR) | 5(19.2)  0(0-0.03) | 2(12.5)  0(0) | 3(18.8)  0(0) | 0  0(0) | 1(25)  0(0-0.75) |
| **Can f 3**  n (%),  median (IQR) | 2(7.7)  0(0) | 1(6.3)  0(0) | 4(25)  0(0-0.49) | 0  0(0) | 0  0(0) |
| **Equ c 3**  n (%),  median (IQR) | 4(15.4)  0(0) | 2(12.5)  0(0) | 5(31.3)  0(0-0.66) | 1(12.5)  0(0) | 0  0(0) |
| **Gal d 5**  n (%),  median (IQR) | 8(30.8)  0(0-0.7) | 1(6.3)  0(0) | 5(31.3)  0(0-0.46) | 0  0(0-0.07) | 1(25)  0(0-0.85) |

*IQR: Interquartile Range

**SD: Standard deviation

n: number, AM: allergen molecule, CM: cow’s milk, SA: serum albumin
